# Stepwise endoscopic management of diospyrobezoar-induced small bowel obstruction using long-cap-assisted suction and balloon catheter

**DOI:** 10.1055/a-2802-4898

**Published:** 2026-02-27

**Authors:** Nobutaka Doba, Kosuke Shibayama, Shinzo Abe, Daiki Sakuma, Masanobu Someya, Kazuto Komatsu, Shin Maeda

**Affiliations:** 1 36998 Department of Gastroenterology, Yokosuka City Hospital, Yokosuka, Japan; 2 Department of Gastroenterology, Yokohama City University Graduate School of Medicine, Yokohama, Japan

**Fig. 1:**
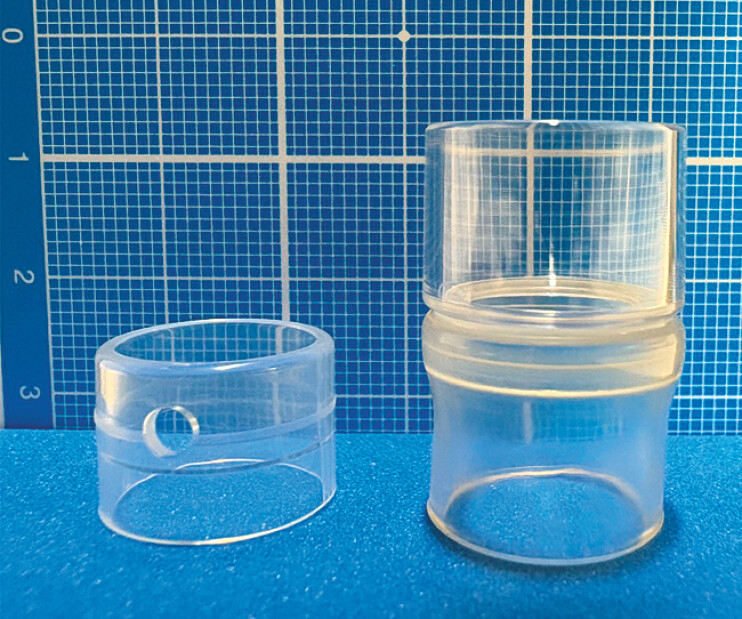
Short versus long transparent cap. Short transparent cap (left) and long transparent cap (right). The long cap protrudes 12 mm beyond the endoscope tip and has a larger diameter and inner lumen, facilitating suction and retrieval.

Small bowel obstruction caused by diospyrobezoars is often difficult to manage endoscopically, and surgical intervention is therefore frequently selected 1 2 3 . We present a case of small bowel obstruction due to ileal diospyrobezoars that was successfully treated using a stepwise endoscopic strategy combining long-cap-assisted suction and an endoscopic retrograde cholangiopancreatography (ERCP) balloon catheter ( Fig. 1 ).

**Fig. 2:**
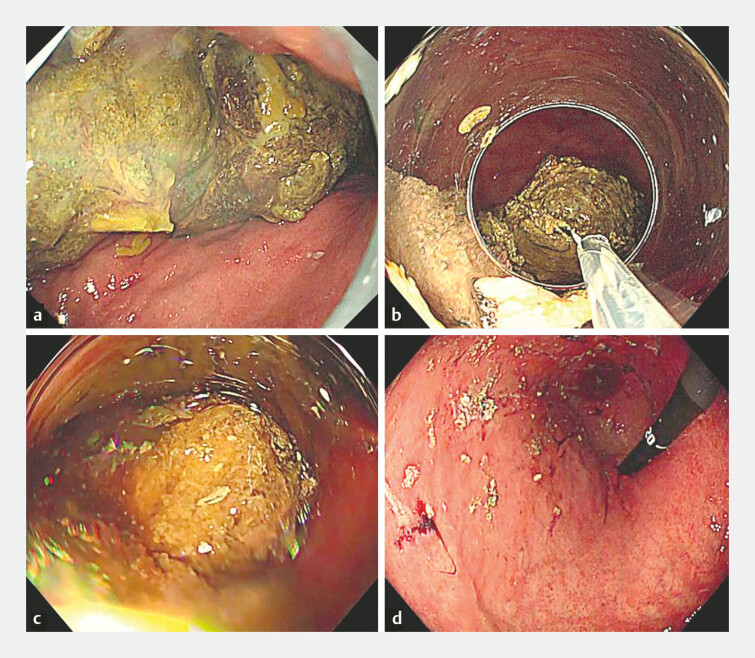
Endoscopic treatment of gastric bezoars. **a** A pre-treatment endoscopic view showing multiple gastric bezoars larger than 5 cm. **b** Fragmentation of gastric bezoars using a guidewire fashioned into a snare and a conventional snare. **c** Retrieval of gastric bezoars using long-cap-assisted suction. **d** A post-treatment endoscopic view showing no clinically significant residual gastric bezoars.

To prevent further migration of gastric bezoars into the small bowel, the endoscopic removal of the gastric bezoars was attempted first. Multiple large gastric bezoars were identified in the stomach and fragmented using a guidewire fashioned into a snare and a conventional snare ( Fig. 2 **a, b** , Video 1 ). When grasping was ineffective, the bezoar was pressed against the rim of the long cap to facilitate fragmentation 4 ( Video 1 ). Retrieval with a net was difficult because of physiological esophageal narrowing at the esophageal hiatus and tracheal bifurcation; however, long-cap–assisted suction enabled effective removal, with no clinically significant residual gastric bezoars 5 ( Fig. 2 **c, d** , Video 1 ).

Stepwise endoscopic treatment of diospyrobezoars using long cap-assisted suction and a balloon catheter.Video 1

**Fig. 3:**
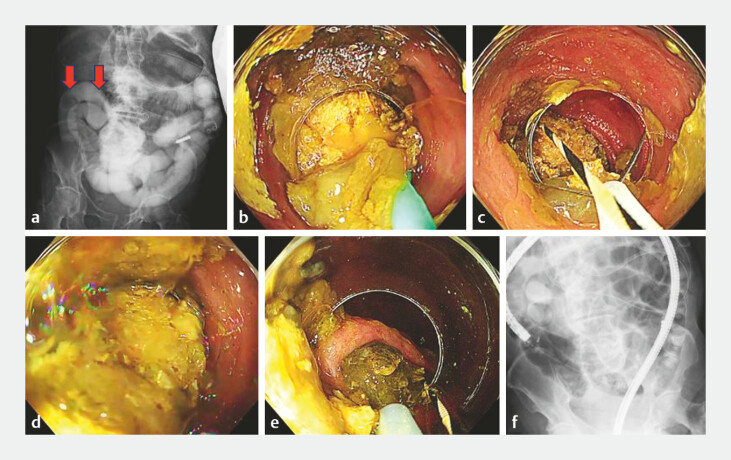
Endoscopic and fluoroscopic management of ileal bezoars. **a** The pre-treatment fluoroscopic image demonstrating two bezoars in the ileum. Arrows indicate the bezoars. **b** An endoscopic view of the more distal ileal bezoar being mobilized within the ileum using an ERCP balloon catheter. **c** Because of sharp ileal angulation and the large size of the bezoar, advancement into the colon was not feasible; therefore, the bezoar was fragmented within the ileum using a snare. **d** Retrieval of the fragmented bezoar into the colon using long-cap-assisted suction. **e** The second, smaller ileal bezoar was mobilized with a balloon catheter and retrieved to the ileocecal valve using a snare. **f** The post-treatment fluoroscopic image confirming the absence of residual bezoars in the ileum.

Endoscopic treatment of the ileal bezoars causing the obstruction was then performed. Fluoroscopy revealed two bezoars in the ileum ( Fig. 3 **a** ). Because direct endoscopic access was difficult, the more distal bezoar was mobilized to the ileal flexure using an ERCP balloon catheter ( Fig. 3 **b** , Video 1 ). Owing to sharp ileal angulation and the large size of the bezoar, advancement into the colon was not feasible; therefore, it was repositioned, fragmented within the ileum, and retrieved using long-capassisted suction ( Fig. 3 **c, d** , Video 1 ). The second, smaller ileal bezoar was similarly mobilized, grasped with a snare, and retrieved to the level of the ileocecal valve ( Fig. 3 **e** , Video 1 ). The final fluoroscopic and endoscopic evaluation confirmed that no clinically significant residual ileal bezoars remained, with resolution of the small bowel obstruction ( Fig. 3 **f** , Video 1 ).

This stepwise endoscopic approach represents a practical and minimally invasive option for managing diospyrobezoar-induced small bowel obstruction and may help avoid surgical intervention.

Endoscopy_UCTN_Code_TTT_1AQ_2AH
